# Localization of Fibrinogen in the Vasculo-Astrocyte Interface after Cortical Contusion Injury in Mice

**DOI:** 10.3390/brainsci7070077

**Published:** 2017-07-06

**Authors:** Nino Muradashvili, Suresh C. Tyagi, David Lominadze

**Affiliations:** Department of Physiology, University of Louisville, School of Medicine, Louisville, KY 40202, USA; n0mura01@louisville.edu (N.M.); s0tyag01@louisville.edu (S.C.T.)

**Keywords:** astrocyte endfeet, cerebral vessels, neuronal degeneration, short-term memory

## Abstract

Besides causing neuronal damage, traumatic brain injury (TBI) is involved in memory reduction, which can be a result of alterations in vasculo-neuronal interactions. Inflammation following TBI is involved in elevation of blood content of fibrinogen (Fg), which is known to enhance cerebrovascular permeability, and thus, enhance its deposition in extravascular space. However, the localization of Fg in the extravascular space and its possible interaction with nonvascular cells are not clear. The localization of Fg deposition in the extravascular space was defined in brain samples of mice after cortical contusion injury (CCI) and sham-operation (control) using immunohistochemistry and laser-scanning confocal microscopy. Memory changes were assessed with new object recognition and Y-maze tests. Data showed a greater deposition of Fg in the vascular and astrocyte endfeet interface in mice with CCI than in control animals. This effect was accompanied by enhanced neuronal degeneration and reduction in short-term memory in mice with CCI. Thus, our results suggest that CCI induces increased deposition of Fg in the vasculo-astrocyte interface, and is accompanied by neuronal degeneration, which may result in reduction of short-term memory.

## 1. Introduction

Traumatic brain injury (TBI) is one of the most common neurological disorders leading to memory impairment, causing reduction in short-term memory, in particular [1]. The harmful effects of TBI occur during primary injury induced by a mechanical force resulting in compression and physical damage of vessels and neurons, and after secondary complications (e.g., inflammation) that follow the injury. One of the complications that occurs after inflammation is increased vascular permeability [2]. We showed that TBI-induced increase in cerebrovascular permeability leads to an enhanced movement of proteins across vessel walls located at least 200 μm away from the injury perimeter [3]. We call this area the “injury penumbra”. In particular, we have shown that high molecular weight glycoprotein fibrinogen (Fg), which is one of the inflammatory markers, traverses the cerebrovascular wall after cortical contusion injury (CCI) [3]. The level of injury we induced did not result in severe vascular ruptures (defined by absence of large deposits of fibrin in extravascular space) in the “injury penumbra”, and was characterized as “mild” CCI by others [4]. We found that deposition of Fg in the extravascular space was enhanced in mice after CCI [3]. However, localization of deposited Fg, and therefore its possible interaction with nonvascular cells in the brain, are not known. 

It is known that increased presence of Fg-containing plaques leads to severe impairment in memory in diseases such as vascular dementia [5] and Alzheimer’s disease [5,6,7,8]. Although amyloid beta protein is known to be associated with Alzheimer’s disease [8], a greater role of cellular prion protein (PrP^C^) in memory reduction has been shown [9,10]. It was found that Fg interacts with non-digested PrP^Sc^ [11]. Our previous data indicated an increased formation of Fg-PrP^C^ complex and its association with reduction of short-term memory after CCI [3]. However, it is not clear where the Fg-containing protein complex can be formed, and how its location can affect neurons. It has been shown that stub wound injury (SWI)-induced hemorrhage containing fibrin and exogenously delivered Fg/fibrin induce mobilization of astrocytes 3 days after injury [12]. Whilst astrocyte activation by fibrin has been shown around traumatically damaged blood vessels, it is not yet known where Fg/fibrin is deposited perivascularly, or what effect this has on astrocyte activation [12].

Since astrocytes are the main connective links between the cerebral vessels and neurons, we hypothesized that during inflammatory pathology (e.g., TBI), after crossing the blood vessel wall, Fg deposits at the interface between the blood vessel and the astrocyte endfeet. The place of Fg deposition in the extravascular space is important, as it may cause activation of existing astrocytes and thus result in vasculo-neuronal physical, and possibly functional, uncoupling, leading to neuronal dysfunction and thus reduction in memory. Therefore, in the present study, we investigated the location of Fg deposition 14 days after CCI when Fg can appear in the extravascular space only via crossing intact vascular walls. 

## 2. Materials and Methods

All animal procedures for the study were reviewed and approved by the Institutional Animal Care and Use Committee of the University of Louisville in accordance with National Institute of Health Guidelines for animal research.

Polyclonal rabbit anti-human Fg antibody was obtained from Dako (Carpinteria, CA, USA). Anti-NeuN monoclonal antibody (cloneA60), Anti-glial fibrillary acidic protein (GFAP) cocktail (SMI-22), and Fluoro-Jade C were purchased from EMD Millipore/Life Science (Billerica, MA, USA). Secondary antibodies conjugated with Alexa-Fluor 488, Alexa-Fluor 594, or Alexa-Fluor 647 were from Invitrogen (Carlsbad, CA, USA). 4,6-diamidino-2-phenyl-indole HCl (DAPI) was from Santa Cruz Biotechnology (Santa Cruz, CA, USA). Normal Donkey Serum (NDS) was purchased from Jackson ImmunoResearch (West Grove, PA, USA). Texas Red-conjugated Lycopersicon Esculentum agglutinin (TXR-LEA) tomato lectin was from Vector laboratories (Burlingame, CA, USA). 

Male C57BL/6J wild-type (WT) mice were purchased from the Jackson Laboratory (Bar Harbor, ME) and bred in-house. Twelve-week old, 26–30 g mice were anesthetized with 2.5% isoflurane and placed in a stereotaxic frame (Kopf, Tujunga, CA, USA). The shaved skull was exposed, and a 4 mm diameter craniotomy was made at −2.5 mm bregma and 2.75 mm lateral to the midline over the left hemisphere without disturbing the dura mater [3,13]. The impactor device (TBI 0310, Precision Systems & Instrumentation, Fairfax Station, VA, USA) with 2 mm diameter flat tip was set to deliver an impact (0.5 mm impact depth, 3.5 m/s velocity, 500 ms) to the cortical surface that would create a “mild” TBI [4]. After the impact, the skull cap was replaced using Surgiseal (Johnson & Johnson) and the skin was sutured. Body temperature was maintained at 37 °C by keeping mice on a heating pad till their full recovery. In sham-operated (control) group, everything was repeated except the actual impact.

It has been shown that after an inflammatory insult, the blood level of Fg increases starting from day 4 and reaches its maximal levels in 12–16 days [14]. Therefore, data presented in this study were obtained 14 days after CCI.

### 2.1. Immunohistochemistry

Fourteen days after CCI and sham-operation, anesthetized mice were infused with TXR-LEA via carotid cannulation to fluorescently label the intravascular endothelial surface [3,13]. Animals were sacrificed with pentobarbital overdose, and were then immediately infused with phosphate buffered saline (PBS) followed with 4% paraformaldehyde (PFA) solution through the left ventricle [3]. The brain was gently removed after opening the cranium and placed in 4% PFA solution overnight. Then, it was kept in 30% Sucrose for 3 days prior to the sample preparation for immunohistochemistry [3,13]. Brain samples were mounted in protective matrix (Polyscience, Inc., Warrington, PA, USA) and cryosectioned on the coronal plane using a Leica CM 1850 Cryocut (Bannockburn, IL, USA). Twenty five µm thick slices were thaw-mounted on charged microscope slides (VWR, West Chester, PA, USA). 

For further processing, samples were warmed and the mounting matrix was removed [3]. The sections were post-fixed in ice-cold 100% methanol for 10 min, washed in Tris buffered saline (TBS) and blocked for non-specific epitope binding in 0.1% TritonX-100 TBS (TBS-T), 0.5% bovine serum albumin (BSA) and 10% normal donkey serum (NDS) for 1 h at RT. Primary antibodies against NeuN (dilution 1:100), GFAP (dilution 1:100), and Fg (dilution 1:200), were applied to the brain slices overnight at 4 °C. After washing, respective fluorescent dye-conjugated secondary antibodies (dilution 1:200) were applied to the brain slices for 1 h at RT. Slides were washed in TBS-T and TBS solutions sequentially, air dried, and covered with a glass coverslip. 

In another series of experiments, for Fluoro-Jade C staining, the mouse brain tissue preparation was done according to the manufacturer’s (EMD Millipore/Life Science, Billerica, MA, USA) recommendation. Briefly, slides were incubated in 80% ethanol containing 1% sodium hydroxide, then rinsed for 2 min in distilled water after rinsing in 70% ethanol followed by incubation in 0.06% potassium permanganate for 10 min. After rinsing in distilled water, slides were transferred to 0.0001% Fluoro-Jade C solution for 10 min and then thoroughly washed in distilled water. The slides were air dried at 50 °C for 5 min, cleared in xylene for 1 min and covered with glass coverslips.

Brain samples were observed with a laser-scanning confocal microscope (Olympus FluoView1000, with objective PlanApo-60x/1.40 oil). Off-line image analysis software (Image-Pro Plus 7.0, Media Cybernetics; Bethesda, MD, USA) was used to assess expression of Fluoro-Jade C, NeuN, GFAP, and deposition of Fg. For each experimental group, 3–4 brain slices were analyzed. Fluorescence intensities of Fluoro-Jade C and NeuN were measured in 5 randomly placed constant size areas of interest (AOI). The results were averaged for each experimental group and values were presented as fluorescence intensity units (FIU).

To define the level of Fg deposition and its location in the extravascular space a total of 4 focal planes of the tissue samples were analyzed. Distance between the analyzed focal planes was set at 0.5 µm. Fluorescence intensity of Fg was measured in each image and was normalized by the length of the respective vascular segment. Data were presented as FIU. Co-localization of GFAP and Fg in brain tissue was assessed by measuring the number of spots generated in result of co-localization of respective colors in images formed after deconvolution of an original image as described earlier [15]. Obtained results were averaged for each experimental group.

### 2.2. Short-Term Memory Assessment 

A novel object recognition test (NORT) was used to assess visual short-term memory [16], and was performed as described previously [3,17]. Briefly, after acclimatization, mice were trained during two days in the test box for 10 min twice a day. On the day of the test, each mouse was placed in the middle of the box opposite two similar objects and allowed to investigate the objects for 5 min. After an hour, one of the objects was replaced with a differently shaped object, and the animal was returned to the box for 3 min. The Top Scan behavioral analyzing system (Version 3.00 by CleverSys. Inc.; Reston, VA, USA) was used to record and analyze the mouse behavior. Discrimination ratio (DR, time spent at the novel object/time spent at both objects) was calculated. Lower DR indicates an impairment of memory. 

**Figure 1:**
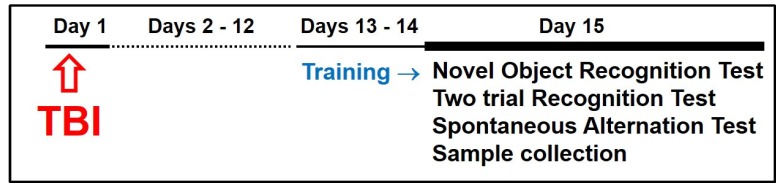
Experimental timeline.

Using a Y-maze, a spontaneous alternation test was performed to assess spatial working short-term memory [18] as described [16,18,19,20]. A separate set of age-matched animals were placed in the middle of the Y-maze with all three arms open. All arm entries were sequentially recorded for 8 min. The percentage of alternation was calculated as the ratio of actual alternations (subsequent entries into three arms on overlapping triplet sets) to possible alternations (the total number of arm entries minus two), and multiplied by 100 [16,21]. A two-trial recognition test was conducted as described [19,20]. Each mouse from yet another set of age-matched animals was placed in one of the three arms (start arm) of the Y-maze, while one of the remaining two arms was blocked. The mouse was allowed to explore the maze for 10 min. After an hour, the mouse was returned to the maze with all arms opened and its behavior was recorded for 5 min. The choice of first-entry and the number of all entries to each arm were recorded. To define possible changes in short-term memory, a discrimination index (DI) was calculated as the number of entries to the novel arm, and expressed as a percentage of the total number of entries to all arms. This variable defines place recognition memory [22], and can also be called spatial discrimination memory [23]. The number of mice in each group that entered to the novel arm first is presented as a percent of all tested mice in the group. This variable would indicate discrimination memory [23]. The overall experimental timeline is presented in Figure 1.

All data are expressed as mean ± SEM. The experimental groups were compared by one-way ANOVA. Differences were considered significant if *p* < 0.05.

## 3. Results 

**Figure 2:**
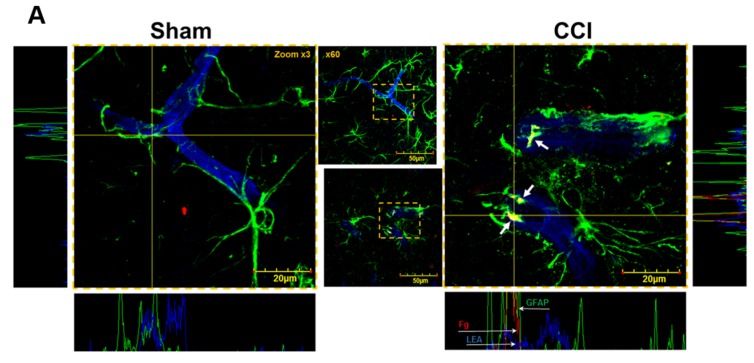

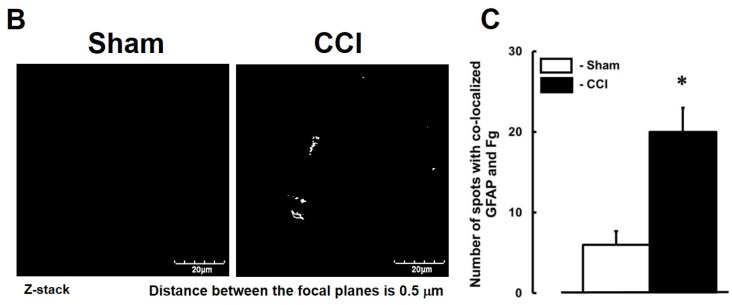
Deposition of fibrinogen (Fg) in vasculo-astrocyte interface after cortical contusion injury (CCI) in mice. (**A**) Examples of astrocytes and their endfeet surrounding vessels and Fg (red) deposited in the extravascular space 14 days after sham-operation (sham) and CCI. Astrocytes are identified by expression of glial fibrillary acidic protein (GFAP, green) and vascular endothelium is defined by Lycopersicon Esculentum agglutinin tomato lectin (LEA, blue). Images of each area of interest were collected by acquiring at least 4 focal planes with increment of 0.5 µm to form a final z-stack image. The highlighted areas were enlarged 3-times (side images). Short white arrows point to Fg and astrocyte endfeet co-localization (shown in yellow). Placing the Image Pro-plus software’s line profile probes (horizontal and vertical yellow lines) on enlarged images, we obtained fluorescence intensity profiles along the X (panels are shown at the bottom of the respective enlarged images) and Y (panels are shown on the sides of the respective enlarged images) axis. Fluorescence intensity profiles show that at the area of co-localizations (shown in yellow), Fg (red) was located between the vessels (blue) and astrocyte endfeet (green). In the right horizontal panel, thin and long white arrows pointing to a vessel (blue), Fg (red), and astrocyte labeled with GFP (green) in the same vertical plane clearly show Fg between the vessel and astrocyte; (**B**) Shown are examples of z-stack images of spot co-localization after deconvolution of original images of brain samples from mice with sham-operation) or with CCI); (**C**) Average number of co-localized Fg and GFAP spots calculated after deconvolution of experimental stack images are presented for each experimental group. * *p* < 0.05 vs. Sham. *n* = 4.

Immunohistochemistry analysis showed that Fg deposition was increased (61.1 ± 13.5 FIU; *n* = 4) in the extravascular space after CCI compared to that (6.7 ± 0.6 FIU; *n* = 4) in brains of sham-operated mice. We found that deposition of Fg occurred mainly in the vasculo-astrocyte endfeet interface (Figure 2A). Moreover, Fg deposited in the extravascular space was located between the vessel and astrocyte endfeet, predominantly co-localized with the astrocyte endfeet (Figure 2A). Significant changes in astrocyte shape were also noticeable in brain samples of mice after CCI: more astrocytes were visibly swollen indicating their activation (Figure 2A). It is noticeable that there are a few non-activated astrocytes that are not co-localized with deposited Fg (Figure 2A (CCI), bottom part of the image). Higher up on the image (right side), there is an activated astrocyte also not associated with deposited Fg. As the latter can be a result of global activation of astrocytes due to CCI, our data suggest that astrocytic activation can be attributed by the presence of Fg in the extravascular space.

**Figure 3:**
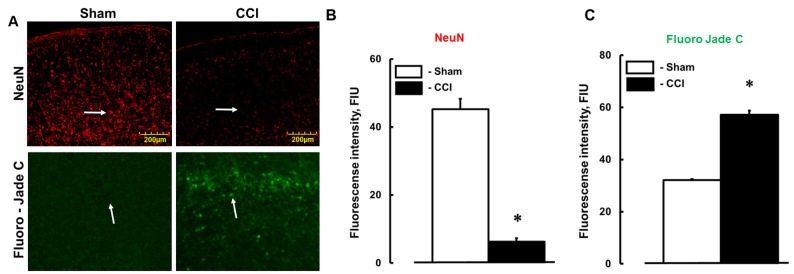
Neuronal degeneration 14 days after cortical contusion injury (CCI) in mice (**A**) Examples of expression of neuronal marker NeuN (red, upper row) and marker of damaged neurons Fluoro-Jade C (green, lower row) in mouse brain samples collected 14 days after sham-operation (sham) and CCI. Summary of (**B**) NeuN and (**C**) Fluoro-Jade C expressions assessed as a measure of their fluorescence intensity in brain samples and presented as fluorescence intensity units (FIU). * *p* < 0.05 vs. WT. *n* = 6.

Expression of NeuN was decreased (Figure 3A,B), while staining of Fluoro-Jade C was increased (Figure 3A,C) in brain samples from mice with injury in comparison to those sample from respective control animals. Although previously we found that overall deposition of Fg/fibrin was increased after CCI in areas farther from the injury [3], loss of NeuN in the present study cannot readily be attributed to Fg/fibrin deposition effect alone.

**Figure 4:**
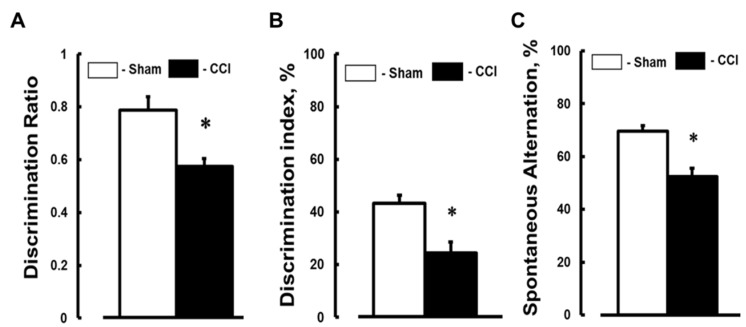
Short-term memory reduction 14 days after cortical contusion injury (CCI) in mice. Results of mice’s short-term memory assessed by (**A**) a novel object recognition test (NORT), and Y-maze (**B**) two trial recognition and (**C**) spontaneous alternation tests. * *p* < 0.05 vs. Sham. *n* = 16.

DR that was defined by NORT was lower in mice with CCI than in the control group (Figure 4A). DI and Spontaneous alternation that were detected with Y-maze tests were also lower in animals with injury than in sham-operated mice (Figure 4B,C, respectively).

## 4. Discussion 

To our knowledge, this is the first study to specifically show the location of Fg that appears in the perivascular space from non-ruptured microvessels after CCI. While previous studies found the presence of Fg/fibrin in amyloid plaques [8,24] and Fg deposits after TBI [25], the first step in their formation and their initial locations (after Fg crosses the vascular wall via transcytosis) were not shown. 

The present data confirm our previous findings that deposition of Fg in the extravascular space is increased after CCI [3]. However, in that study we were not able to define the localization of Fg deposition [3]. In the present study, we demonstrate that Fg is mainly deposited in the existing vasculo-astrocyte endfeet interface. The greater deposition of Fg between the vessels and astrocyte endfeet found in brain samples from mice with CCI compared to that in sham-operated animals can occur only because of a significant increase in cerebrovascular permeability to Fg after TBI [3]. Our finding that astrocytes change shape and become more swollen after CCI suggests that astrocytes are activated as a result of Fg deposition in the vasculo-astrocyte interface. These results are in agreement with data showing that astrocytes were mobilized/activated by Fg/fibrin that appeared in the extravascular space after SWI that caused vascular ruptures [12]. The deposition of Fg between the vessels and astrocyte endfeet suggests their possible interaction. The present results show that extravascular Fg mainly co-localized with activated astrocytes rather than with the vascular wall. Previously, it has been indicated that Fg leaked from damaged vasculature causes astrocyte scar formation [12] and axonal damage [26]. In vitro, astrocytes remove Fg coating from the growth surface, which results in their activation and disappearance (most likely due to their death) [27]. Schachtrup et al. showed that fibrin activates astrocytes by transforming the growth factor beta (TGF-β) receptor pathway, and promotes astrocyte scar formation after vascular rupture resulting from SWI [12]. These findings indicate the possibility of a strong interactive association between Fg and astrocytes, and suggest that Fg may directly activate inflammatory responses [12]. It is known that astrocytes contain intercellular adhesion molecule-1 (ICAM-1) [28], which has a high binding affinity for Fg [29]. It is possible that after crossing the vascular wall, Fg binds astrocyte ICAM-1 and results in astrocyte activation. Most importantly, the appearance of Fg in the vasculo-astrocyte interface leads to a physical detachment of astrocyte endfeet from the vessel. This disrupted connection of astrocyte endfeet with the vessel can lead to the deprivation of neurons of necessary nutrients, resulting in neuronal degeneration and their dysfunction. This would suggest that Fg-induced vasculo-astrocyte physical uncoupling results in vasculo-astrocyte and, as a result, vasculo-neuronal functional uncoupling. Indeed, our findings of the localization of Fg deposition in the extravascular space were accompanied by CCI-induced neuronal degeneration in mice 14 days after the injury. Furthermore, these effects were found along with reduction in short-term memory assessed with NORT and Y-maze tests. Thus, our data indicate that enhanced deposition of Fg in the vasculo-astrocyte interface was accompanied by neuronal denegation and reduction in memory after CCI suggesting a possible functional connection between these events.

Here we showed that after crossing the wall of non-damaged cerebral vessels 14 days after mild brain injury when vascular ruptures are minimal, Fg deposits in the existing vasculo-astrocyte endfeet interface. Being in close proximity to astrocytes, Fg may activate them and result in further mobilization of astrocytes to the location of Fg deposition, as shown by Schachtrup et al. [12].

In conclusion, we present data that support the new hypothesis that after CCI, inflammation-induced increases in the blood level of Fg [3] enhances cerebrovascular permeability in the “injury penumbra”, even 14 days after the injury [3]. This leads to translocation of Fg from inflamed microvessels to the extravascular space of the affected brain. Fg deposits in the vasculo-astrocyte endfeet interface and possibly binds to astrocytes via their receptor, ICAM-1. This possible binding to Fg and trauma-induced inflammation lead to activation of astrocytes and astrocyte-neuronal functional uncoupling. The presence of Fg in the extravascular space, and particularly between the vessel and astrocyte endfeet, causes vasculo-astrocyte physical and, apparently, functional uncoupling. In turn, this vasculo-astrocyte uncoupling may be involved in the development of neuronal dysfunction and, as a result, in the short-term memory reduction seen in the present study. In addition, our previous work showed that the immobilized Fg can bind to PrP^C^ [3,20] and/or amyloid beta [17,20], forming fibrin-containing complexes, which are highly resistant to degradation [8,30], and could lead to formation of protein plaques. The important finding of this study is that Fg/fibrin-containing protein complex formations can only be initiated in the vasculo-astrocyte endfeet interface, and these complexes do not have a direct interaction (at least initially) with neurons. Thus, our data demonstrate an initial localization Fg/fibrin-containing complexes and a possible path of their action during various inflammatory cerebrovascular pathologies and particularly after mild TBI. Therefore, therapeutically targeting enhanced cerebrovascular permeability to diminish the formation of the Fg/fibrin-containing complex seems to be the most appropriate step after inflammatory cerebrovascular diseases resulting in cognitive impairment.
